# Simultaneous Cardioflow Optimisation to facilitate haemodynamic recovery after rapid ventricular pacing during transcatheter aortic valve implantation: a case series

**DOI:** 10.1093/ehjcr/ytaf089

**Published:** 2025-02-24

**Authors:** Rami Jubeh, Elad Asher, Danny Dvir, Michael Glikson, Sharon Bruoha

**Affiliations:** Jesselson Integrated Heart Center, The Eisenberg R&D Authority, Shaare Zedek Medical Center and Faculty of Medicine, Hebrew University of Jerusalem, Shmuel Bite 12, 9103102 Jerusalem, Israel; Jesselson Integrated Heart Center, The Eisenberg R&D Authority, Shaare Zedek Medical Center and Faculty of Medicine, Hebrew University of Jerusalem, Shmuel Bite 12, 9103102 Jerusalem, Israel; Jesselson Integrated Heart Center, The Eisenberg R&D Authority, Shaare Zedek Medical Center and Faculty of Medicine, Hebrew University of Jerusalem, Shmuel Bite 12, 9103102 Jerusalem, Israel; Jesselson Integrated Heart Center, The Eisenberg R&D Authority, Shaare Zedek Medical Center and Faculty of Medicine, Hebrew University of Jerusalem, Shmuel Bite 12, 9103102 Jerusalem, Israel; Department of Cardiology, Barzilai University Medical Center and Faculty of Health Sciences, Ben-Gurion University of the Negev, Hahistadrut 2, 7830604 Ashkelon, Israel

**Keywords:** Severe LM stenosis, Aortic stenosis, Left ventricular dysfunction, Rapid ventricular pacing, TAVR, Case series

## Abstract

**Background:**

‘Simultaneous CardioFlow optimization’ represents an innovative technique that effectively enhances post-pacing haemodynamic recovery during transcatheter aortic valve replacement. This technique was applied in two high-risk patients presenting with severe left main trunk stenosis with concomitant severe aortic stenosis (AS) and left ventricular dysfunction (LVD).

**Case summary:**

An 86-year-old and a 96-year-old patient were admitted with myocardial infarction. Angiography revealed severe left main trunk stenosis, while echocardiography demonstrated significant LVD and severe AS. Both cases were evaluated by the institutional multidisciplinary heart team. Considering the high surgical risk, a percutaneous management strategy was contemplated.

**Discussion:**

We postulated that performing left main trunk balloon angioplasty during rapid ventricular pacing would be better tolerated, compared with conventional balloon angioplasty, given the low coronary perfusion pressure at this stage. Additionally, coronary balloon angioplasty is expected to enhance coronary flow in the immediate post-pacing period, thus facilitating haemodynamic recovery. Both patients exhibited prompt improvement in hemodynamics following rapid ventricular pacing and had uncomplicated hospital stays. In patients with significant left main trunk stenosis accompanied by severe AS and concomitant left ventricular dysfunction, ‘simultaneous CardioFlow optimisation’ facilitates post-pacing haemodynamic recovery.

**Learning points**

- Left main coronary artery (LMCA) stenosis can hinder the recovery of cardiac function and haemodynamics in patients with impaired LV function and severe aortic stenosis undergoing rapid ventricular pacing during transcatheter aortic valve replacement.
- Simultaneous CardioFlow Optimization involves concurrent LMCA balloon angioplasty and balloon aortic valvuloplasty/transcatheter aortic valve replacement during rapid ventricular pacing to enhance post-pacing cardiac recovery.

## Introduction

Aortic stenosis (AS) and coronary artery disease (CAD) share common risk factors and pathophysiologic pathways, leading to their frequent co-existence.^1^ Traditionally, surgical aortic valve replacement combined with coronary artery bypass grafting was the only available treatment option for patients with both conditions and acceptable surgical risk. However, in recent years, transcatheter aortic valve replacement (TAVR) has emerged as the procedure of choice for aortic valve implantation in a substantial subset of patients with severe AS and significant CAD regardless of surgical risk, allowing for effective percutaneous management.^2,3^ Accordingly, contemporary guidelines provide a class IIa recommendation for revascularization with percutaneous coronary intervention (PCI) in patients with a primary indication to undergo TAVR and coronary stenosis >70% in proximal segments.^3^ Nevertheless, data on the optimal management strategy in patients with severe left main coronary artery (LMCA) stenosis and significant left ventricular dysfunction (LVD) undergoing TAVR remain limited. The aim of this paper is to propose a novel technique for the concomitant management of severe AS and severe LMCA stenosis to facilitate haemodynamic recovery in the presence of significant LVD after rapid ventricular pacing during TAVR.

## Summary figure

(*A*) SCO during BAV; (*B*) SCO during TAVR; Arrowhead’s, balloon across aortic valve; star, guiding catheter; bold arrow, coronary balloon inflated in the LMCA; double arrow, coronary wire in LCx; triple arrow, coronary wire in LAD; square, stiff wire in LV; dot, pacing electrode; AS, aortic stenosis; BAV, balloon aortic valvuloplasty; LAD, left anterior descending; LCx, left circumflex; LMCA, left main coronary artery; LV, left ventricle; LCx, left circumflex; TAVR, transcatheter aortic valve replacement.

**Figure:**
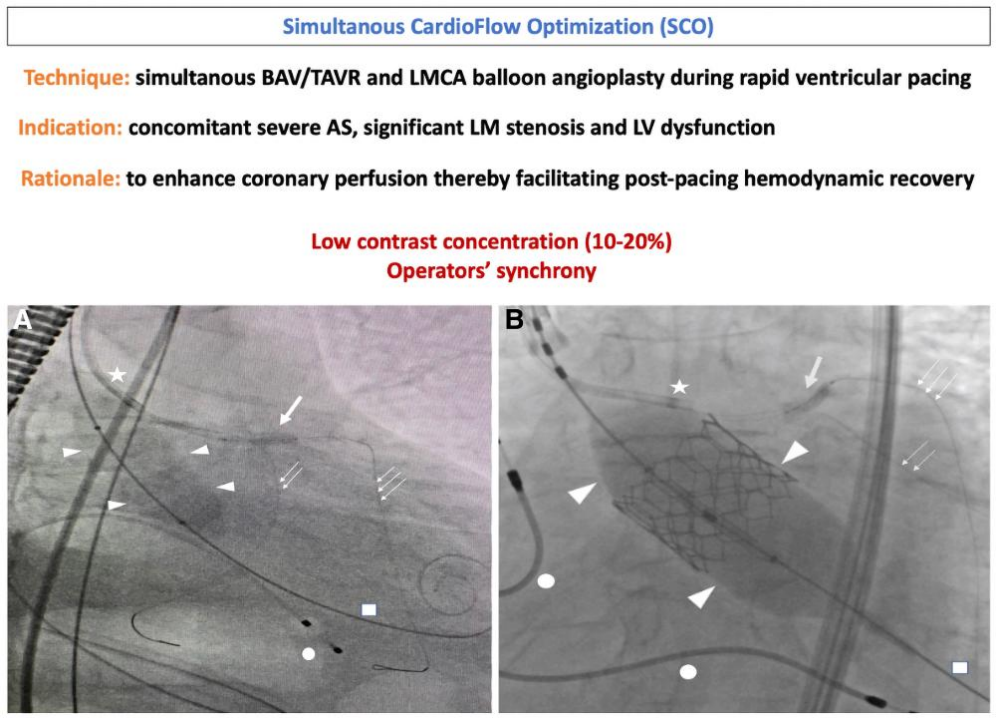


## Case presentation

**Figure 1:**
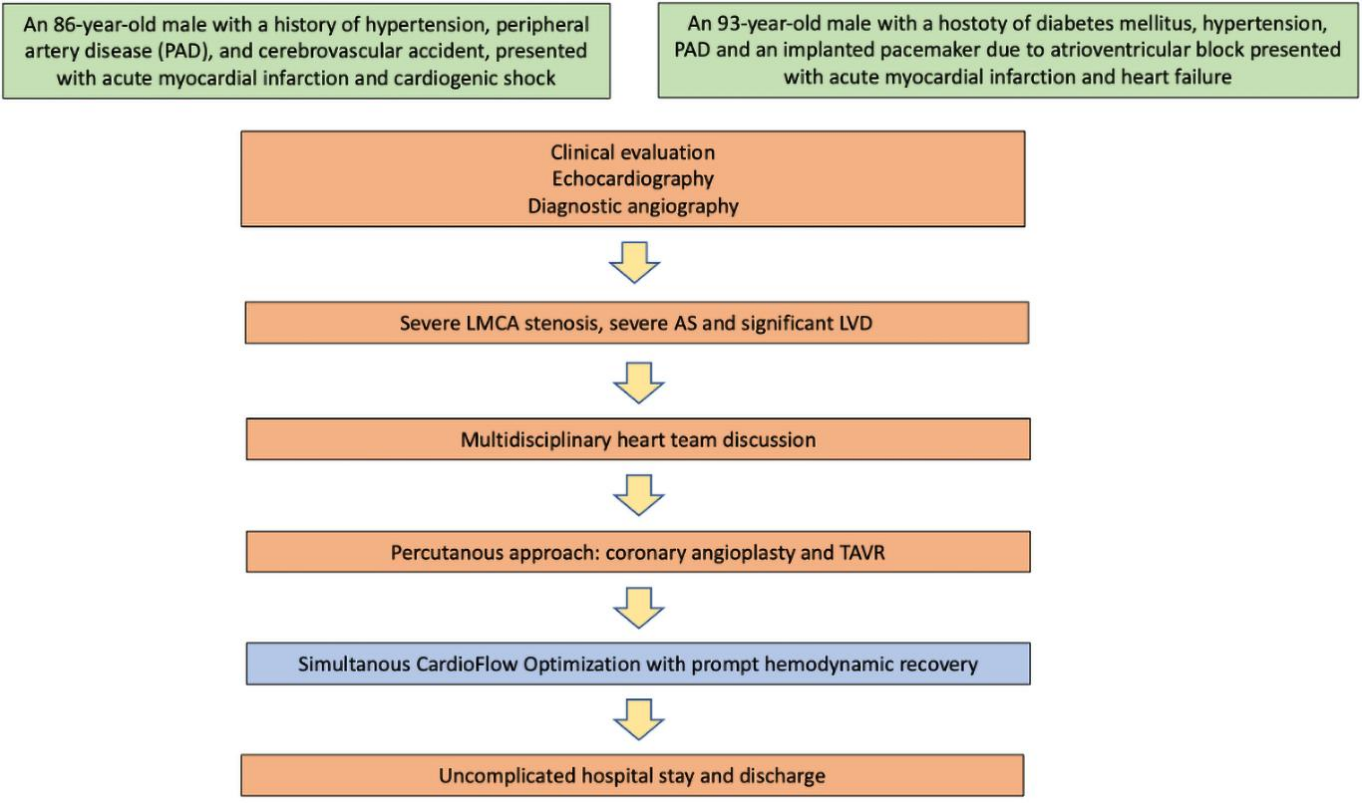
Timeline.

We present two patients who were deemed high-risk for surgery by the institutional multidisciplinary heart team and therefore underwent Simultaneous CardioFlow Optimization (SCO) during transfemoral TAVR (*Figure 1*).

## Patient 1

**Figure 2:**
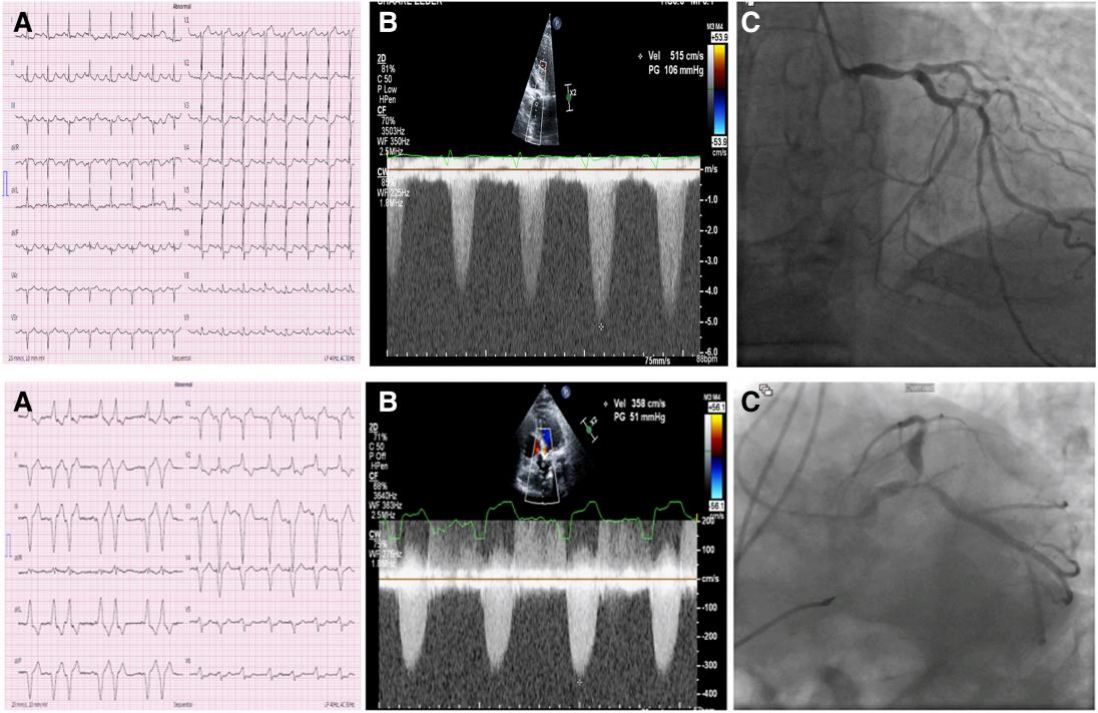
Electrocardiography, echocardiography, and coronary angiography findings in first (upper panel) and second (lower panel) patients. Upper panel (*A*). Electrocardiography showing Sinus tachycardia, diffuse ST-segment depression, and ST-segment elevation in leads aVR and V1; (*B*) CW Doppler of the aortic valve in the apical 5-chamber view showing a jet velocity of 5.15 m/s corresponding with severe AS; (*C*) coronary angiography showing severe stenosis of the distal LMCA and ostia of LAD and LCx coronary arteries (MEDINA 11,1); Lower panel (*A*) Electrocardiography showing sinus rhythm with RV pacing pattern; (*B*) CW Doppler of the aortic valve in the apical 5-chamber view showing a jet velocity of 3.58 m/s; (*C*) coronary angiography showing severe stenosis of the distal LMCA and ostia of LAD and LCx coronary arteries (MEDINA 11,1); CW, continuous wave; AS, aortic stenosis; LMCA, left main coronary artery; LAD, left anterior descending; LCx, left circumflex.

The first case involved an 86-year-old male with a medical history of hypertension, peripheral artery disease (PAD), and a previous cerebrovascular accident, who presented with intermittent chest pain. On physical examination (PE), he had tachycardia, a crescendo-decrescendo systolic murmur, and an absent S2 on cardiac auscultation. His electrocardiogram (ECG) at presentation showed sinus tachycardia, diffuse ST-segment depression, and ST-elevation in lead aVR (*Figure 2*, upper panel). The initial and subsequent high-sensitivity troponin I (hs-cTnI) were 34 and 7000 ng/L, respectively. High-sensitivity troponin I assay was determined in a central laboratory (ARCHITECT STAT hs-cTnI immunoassay) with a 99th percentile reference level of 17 ng/L for females and 35 ng/L for males.

Echocardiography revealed an estimated ejection fraction (EF) of 35%, with a diffusely hypokinetic LV and critical AS, characterized by an aortic valve area (AVA) of 0.4 cm^2^, a mean pressure gradient (PG) of 65 mmHg, and jet velocity of 5.15 m/s (*Figure 2*, upper panel).

Coronary angiography showed mild luminal irregularities in the right coronary artery (RCA) and a tight stenosis in the distal part of the LMCA (*Figure 2*, upper panel). The procedure was aborted for further discussion by the heart team.

Due to the patient’s extremely high surgical risk [Society of Thoracic Surgeons Predicted Risk of Mortality (STS-PROM) score for operative mortality >55% and EuroSCORE II >70%], a hybrid percutaneous approach was considered. This approach involved coronary angioplasty and balloon aortic pre-dilatation followed by TAVR. However, a few hours later, the patient’s condition deteriorated, and he developed cardiogenic shock. He was intubated and required haemodynamic support with high-dose inotropes. After initial stabilisation, the patient was transferred to the catheterization lab.

Given the patient’s critical condition, a pre-procedural computed tomography scan was not performed. Instead, a transoesophageal echocardiography-guided TAVR was conducted. In our opinion, the patient’s critical status justified performing balloon aortic valvuloplasty (BAV) as the initial strategy, followed by LMCA stenting. Moreover, due to the lack of information on the presence and extent of left ventricular outflow tract calcification, we opted not to use a balloon-expandable valve to minimize the risk of annular rupture.

We hypothesized that performing an undersized BAV and LMCA angioplasty simultaneously during rapid pacing (Summary Figure, A)—when cardiac output and coronary blood flow are significantly diminished^4^—could briefly increase the AVA while minimizing the risks of aortic incompetence, annular injury, and haemodynamic instability.^5^ At the same time, LMCA angioplasty could optimize and shorten ischaemic periods immediately after pacing, thus facilitating post-pacing myocardial perfusion and haemodynamic recovery.

**Figure 3:**
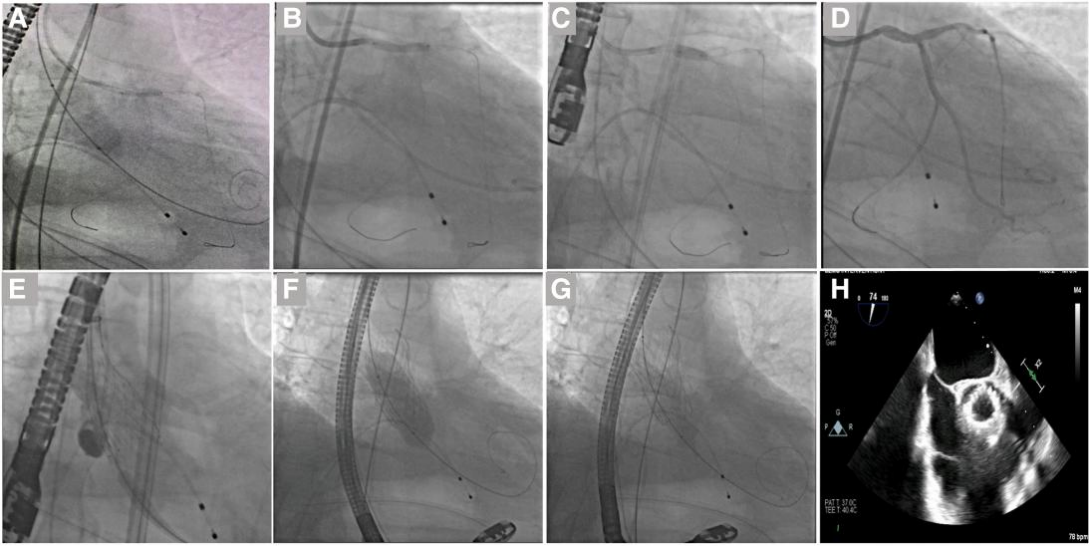
SCO consisting of BAV and LMCA balloon angioplasty during rapid pacing followed by LMCA-LAD provisional stenting and final TAVR with Evolut FX (Medtronic, Minneapolis, MN, USA). (*A*) SCO: LMCA-LAD balloon angioplasty during BAV; (*B*) LMCA stenting; (*C*) POT to the LMCA; (*D*) Post-PCI angiography; (*E*) EVOLUT FX 29 mm deployment, pigtail in non-coronary cusp; (*F*) EVOLUT FX 29 mm balloon post-dilatation; (*G*) final result; (*H*) echocardiographic shot axis view of the aortic prosthesis; BAV, balloon aortic valvuloplasty; LMCA, left main coronary artery; LAD, left anterior descending; NC, non-compliant; PCI, percutaneous coronary intervention; POT, proximal optimization technique; SCO, Simultaneous CardioFlow Optimization; TAVR, transcatheter aortic valve replacement.

The procedure involved advancing an undersized aortic valvuloplasty balloon to the thoracic aorta. Next, a coronary guiding catheter was inserted through the left femoral artery, followed by the wiring of the left anterior descending (LAD) and left circumflex (LCx) coronary arteries. The valvuloplasty balloon was positioned across the aortic valve, and a 3.0 × 15 mm coronary semi-compliant balloon was advanced to the LMCA lesion. A single burst of rapid ventricular pacing at 180 beats per minute (bpm) was delivered, followed by Simultaneous CardioFlow Optimization (SCO)—the concomitant BAV and LMCA balloon angioplasty (*Figure 3A*). The case was concluded with conventional provisional stenting of the LMCA-LAD coronary artery, followed by TAVR (*Figure 3*).

## Patient 2

The second case involved a 93-year-old male who presented with chest pain and shortness of breath. His medical history included diabetes mellitus, hypertension, PAD, and an implanted pacemaker for atrioventricular block. On PE, rales were noted on lung auscultation, and a crescendo-decrescendo murmur with a diminished S2 was evidenced on cardiac auscultation. His ECG revealed a sinus rhythm with a right ventricular (RV) pacing pattern (*Figure 2*, lower panel), and initial hs-cTnI was elevated. A diagnosis of NSTEMI accompanied by heart failure exacerbation was made.

Coronary angiography revealed non-significant disease in the RCA but identified significant proximal LMCA stenosis (*Figure 2* lower panel). Echocardiography showed diffuse hypokinesis with severely reduced LV function (an estimated EF of 30%) and severe low-flow, low-gradient AS, characterized by a stroke volume index of 23 mL/m^2^, AVA of 0.5 cm^2^, a mean PG of 32 mmHg, and a jet velocity of 3.58 m/s. Given the high operative mortality risk (STS-PROM score >10%; EuroSCORE II >55%), we planned a TAVR and concomitant LMCA balloon angioplasty.

**Figure 4:**
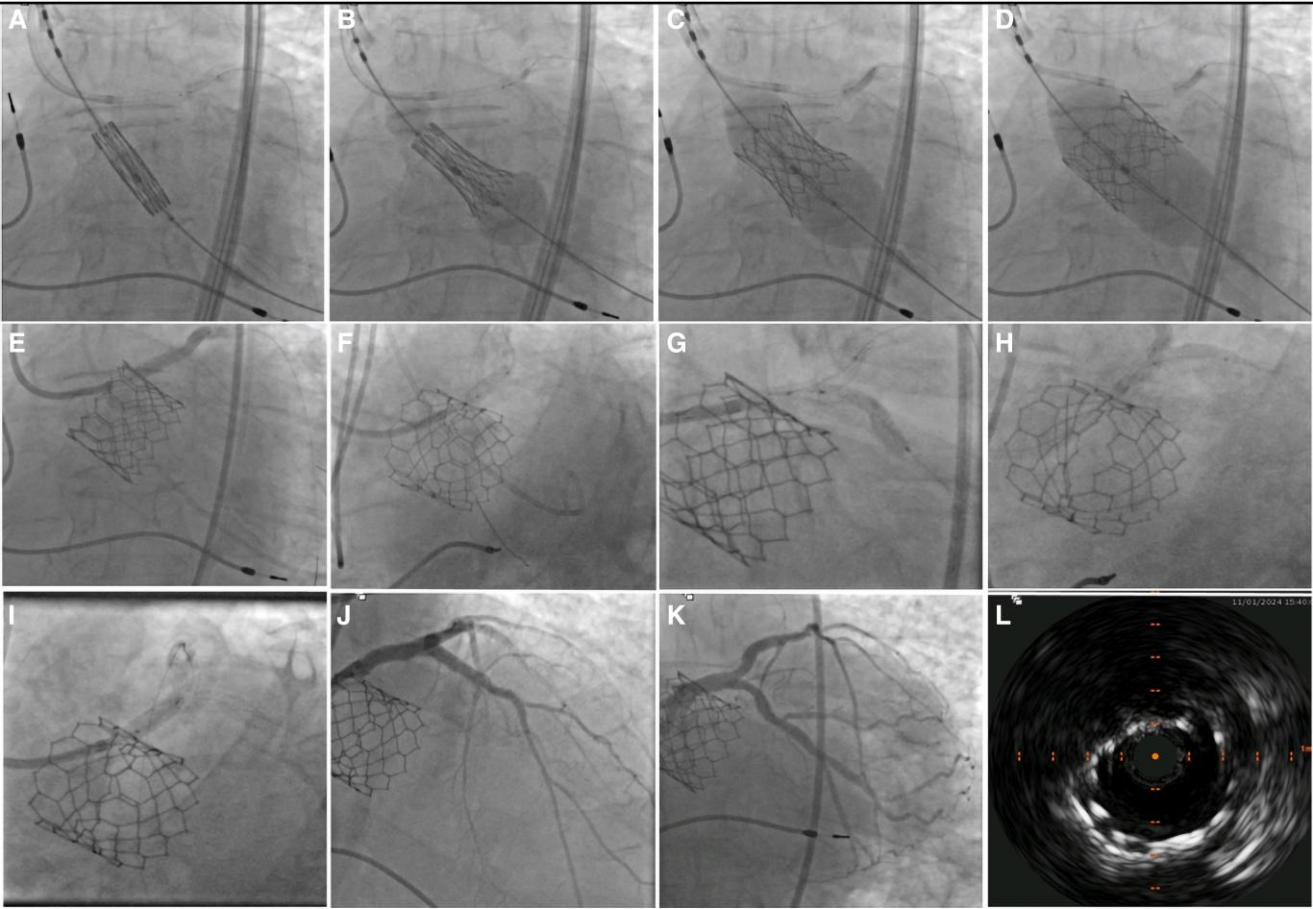
SCO consisting of a SAPIEN 3 (Edwards Lifesciences, Inc., Irvine, CA, USA) TAVR and LMCA balloon angioplasty followed by bifurcation stenting to LAD/LCx. (*A*) Initial position of the coronary balloon in the LMCA-LAD and the SAPIEN 3 29 mm across the aortic valve; (*B–E*) SCO to LMCA-LAD during TAVR; (*F*) POT with a NC balloon; (*G*) placement of DES to LCx (TAP); (*H*) Kissing balloon inflation; (*I*) Re-POT with NC balloon; (*J–K*) Final angiographies; (*L*) IVUS of LMCA stent. DES, drug eluting stent; LAD, left anterior descending; LCx, left circumflex; LMCA, left main coronary artery; NC, non-compliant; POT, proximal optimization technique; SCO, Simultaneous CardioFlow Optimization; TAVR, transcatheter aortic valve replacement; TAP T and protrusion; THV, transcatheter heart valve.

We assumed that LMCA angioplasty would enhance coronary flow and myocardial perfusion, thereby improving post-pacing cardiac contractility and haemodynamic stability. After crossing the aortic valve, the prosthetic valve was positioned in the ascending aorta. This was followed by cannulation of the LMCA artery and coronary wiring. The valve was then advanced to the deployment position, while a 3.0 × 15 mm coronary semi-compliant balloon was positioned across the LMCA stenosis. A single burst of rapid ventricular pacing at 180 bpm was delivered, followed by SCO (Summary Figure, B)—concomitant balloon-expandable TAVR and LMCA balloon angioplasty. The procedure concluded with a conventional T and protrusion bifurcation stenting to the LMCA, LAD, and LCx arteries (*Figure 4*).

Both patients experienced prompt improvement in haemodynamics following rapid ventricular pacing and had an uncomplicated hospital stay.

Importantly, we used a low concentration of contrast (10–20%) to allow for rapid balloon inflation and deflation, in addition to precise operator synchrony.

## Discussion

The optimal timing of PCI in patients with significant CAD, severe AS, and LVD remains unclear due to a lack of contemporary randomized data. One study evaluated the impact of severe AS on short-term outcomes in patients with CAD undergoing PCI (including 6.7% with unprotected LMCA disease) compared with a propensity-matched cohort without AS. The study found similar 30-day mortality rates between patients with and without AS (4.3% vs. 4.7%, *P* = 0.2). However, a significantly higher 30-day mortality rate after PCI was reported in the subgroup with severe AS and severe LVD (EF ≤30%).^6^ Thus, PCI is generally considered safe in patients with severe AS and a low-risk clinical profile, whereas performing PCI in the presence of severe AS and LVD is associated with poorer outcomes. However, only a small number of patients with LMCA stenosis were included in this study.^7^ In contrast, the TAVR-LMCA registry provided a unique opportunity to explore clinical practices and outcomes among 185 patients with severe AS and LMCA stenosis planned for TAVR. Notably, 95% of patients in the registry underwent PCI, primarily in a staged procedure before TAVR (94.8%) rather than concomitantly during TAVR (5.2%). All patients who underwent a hybrid procedure had PCI performed before valve implantation. The remaining 5% underwent LMCA PCI after TAVR in a separate procedure. One-year mortality was similar between patients undergoing planned TAVR plus LMCA PCI and a matched cohort undergoing TAVR without LMCA PCI, suggesting a negligible impact of LMCA PCI on procedural risk. However, the mean EF% among the study participants was 51.5.^8^ Thus, outcomes in patients with concomitant severe AS, LMCA stenosis, and significant LVD remain largely unknown.

In this context, addressing proximal coronary lesions during the short period of reduced coronary flow associated with rapid ventricular pacing required for BAV/TAVR could improve coronary flow and potentially enhance post-pacing LV contraction and haemodynamic recovery, especially in the presence of LV dysfunction. The rationale behind our proposed technique is that performing LMCA balloon angioplasty during the brief period of diminished coronary flow associated with rapid ventricular pacing may be better tolerated than initial isolated LMCA angioplasty.

Importantly, since both of our patients presented with NSTEMI, we assumed that the globally reduced LV function was, at least in part, due to ischaemia and not solely due to the increased afterload associated with severe AS.

In summary, as the population ages, the prevalence of high-risk patients with severe CAD, concomitant AS, and LVD is expected to increase. Consequently, the need for techniques that improve patient safety will become increasingly vital. Our SCO technique may prove to be a valuable tool in managing high-risk patients with complex cardiac conditions.

## Data Availability

The data underlying this article will be shared on reasonable request to the corresponding author.

## Lead author biography

Rami Jubeh, Interventional Cardiologist and Director of Cath Lab Services at Shaare Zedek Medical Center in Jerusalem, Israel, specializes in complex high-risk indicated procedures and chronic total occlusions. Renowned for proficiency in transcatheter aortic valve implantations.

**Consent**: The authors confirm that written consent for the submission and publication of this case report, including images, and associated text, was obtained from the patient in accordance with COPE guidance.
